# Fungistatic Activity of Freshly Killed Termite, *Nasutitermes acajutlae*, Soldiers in the Caribbean

**DOI:** 10.1673/031.007.1401

**Published:** 2007-03-12

**Authors:** Claire A. Fuller

**Affiliations:** Department of Biology, Murray State University, 334 Blackburn Hall, Murray, KY 42071

**Keywords:** Termite, parasitism, competition

## Abstract

Termites may have high exposure to both pathogenic and competitive fungal species. Previous studies have shown anti-fungal properties of the primary components (α-pinene and limonene) of *Nasutitermes* frontal glands that are present on soldiers. In this study, the termite *Nasutitermes acajutlae* (Isoptera: Termitidae) was used to examine if the growth of naturally occurring fungi was inhibited by soldiers, as compared to workers that do not have frontal glands. Soldiers and workers were killed, surface sterilized, and placed on nutrient agar either singly or in combinations with other termites. Time until appearance of fungus and growth of visible fungal colonies was determined. Fungus appeared significantly earlier in cultures with single workers than with single soldiers (P < 0.001). Once fungus appeared, there were no significant differences in growth rate. When worker and/or soldier fluids were combined in one culture, fungal growth appeared later in cultures containing soldiers; growth was significantly slower in colonies with 5 workers and 5 soldiers than in cultures with 5 workers alone; P < 0.001. Finally, growth appeared later in cultures with 5 soldiers than in cultures with one soldier, suggesting a dose-response. Fungi that grew from termites were mostly non-pathogenic, suggesting the anti-fungal properties of soldiers may inhibit both pathogens and competitors.

## Introduction

Animals that live in close social groups can accrue many benefits of sociality such as increased defense against predators, competitors and increased ability to find food. However, one key cost associated with sociality can be an increase in transmission of parasites and pathogens (Schmid-Hempel 1998). Because social insects live in densely populated colonies, encounters between individuals are frequent and often include grooming and trophollaxis. Thus, transmission of diseases among colony members may be high compared to non-social organisms (Rosengaus and Traniello 1997; Schmid-Hempel 1998).

Wood-feeding termites forage in damp wood, often traveling through moist soils and leaf litter to reach foraging sites. They are likely to be highly exposed to pathogens during foraging trips and may bring these microorganisms back to the nest (Rosengaus et al. 2003). Fungi are especially important pathogens of insects and many thrive in the same warm, moist environments as termites. Thus, due to their environment, termites may be especially exposed to pathogenic fungi. Non-pathogenic fungi may also be ecologically important to wood-feeding termites. These termites encounter numerous non-pathogenic fungal degraders. Although some of these fungi may “condition” wood by degrading cellulose, making it more palatable for termites, others are toxic to and/or compete with termites for food (Batra and Batra 1979; Noirot and Noirot-Timothee 1969; Sands 1969). Fungal competitors are especially important for the higher termites (Becker and Lenz, 1976, Batra and Batra 1979). Thus, the ability to suppress microbial growth, especially of fungal pathogens and competitors, could be highly beneficial to termites.

Social insects use a variety of mechanisms to inhibit growth of microbes. For example, ants use behavioral mechanisms such as increased allocation to middens outside of the nest (Hart et al. 2002), glandular secretions (e.g., Beattie et al. 1985, Mackintosh et al. 1995) and cultured bacteria (Currie et al. 1999). Lower termites have also been shown to utilize a number of methods to suppress microbial growth. For example, Rosengaus et al. 1999 found that in *Zootermopsis angusticollis* nests the presence of fungal conidia elicited an alarm behavior. Rosengaus et al. (1998a) found evidence that allogrooming in *Z. angusticollis* decreased pathogen susceptibility and Chen et al. (1998) found that *Coptotermes formosanus* nesting material, composed primarily of fecal material, acts as a fumigant. Fecal material (Rosengaus et al. 1998b) and sternal gland secretions (Rosengaus et al. 2004) of *Z. angusticollis*, were found to inhibit growth of entomophagous fungi (*Metarhizium anisopliae*).

Fewer studies have examined antimicrobial activity of higher termites although these animals account for approx. 85% of isopteran species (Kambhampati and Eggleton 2000). Rosengaus et al. (2000) demonstrated that terpenoids found in the frontal glands of soldiers of the higher termite genus *Nasutitermes* inhibit the germination of the entomophagous fungus, *Metarhizium anisopliae*. They assessed the effect of the main chemical components of the glands. However, workers of *N. costalis* and *N. nigriceps* that were exposed to *M. anisopliae* did not accrue a survival advantage when reared with soldiers, presumably because the cost to workers of feeding soldiers via trophollaxis outweighed the benefit of antifungal secretions of soldier nestmates. It was not determined whether fungistatic activity inhibited non-pathogenic fungi.

Here, the findings of Rosengaus et al. 2000 are extended using the Caribbean termite, *Nasutitermes acajutlae*. The ability of freshly killed *N. acajutlae* to inhibit fungal growth is used to determine the fungistatic activity of termites in the absence of social interactions. Trials are done using a large number (32) of colonies. In these trials, the growth of naturally-occurring fungus is compared in the presence of one dead worker or one dead soldier to determine whether fungus is inhibited by soldiers. However, *N. acajutlae* workers are approx. 3 times larger than soldiers (unpubl. data). Additionally, workers may encounter both pathogenic and non-pathogenic fungi more frequently than soldiers because they ingest wood directly. Therefore, the presence of fungal conidia both on the cuticle and internally, may be a function of body size and exposure, rather than inhibition by soldier frontal glands. These possibilities were tested by examining fungal growth in the presence of a variety of combinations of workers and soldiers. Hypothesis 1 (biomass/exposure) predicts that fungal growth increases with total termite biomass and/or worker number. Hypothesis 2 predicts that growth decreases with the presence of soldiers, whether or not workers are also present. Further, fungal growth should increase with worker number in the absence of soldiers because workers do not have terpenoids. Finally, since Rosengaus et al. (2000) showed an increase in antimicrobial activity with increasing α-pinene and limonene concentration, the effect of the number of soldiers on antifungal properties was examined.

## Methods

### Comparison of fungal growth in the presence of 1 soldier *vs* 1 worker

Termites were collected from 32 colonies on the island of St. John, Virgin Islands, between 28 June and 2 July, 2003. Termites were collected from foraging trails within 2 m of nest cartons by breaking open trails and gently brushing animals and trail material into a plastic container. Termites were transported to the laboratory and sorted by colony. Entomopathogenic fungi are often out-competed by non-pathogenic fungi in culture (R. Humber, USDA ARSEF, pers. comm.), thus all animals were surface sterilized to increase the chance of finding pathogenic fungi. Five soldiers and 5 workers from each colony were killed and surface-sterilized by dipping in a 5.2% sodium hypochlorite solution (Rosengaus and Traniello 1997) for 10 s and rinsing in sterile water. Most termites died during this procedure. Those that didn't were killed by squeezing the prothorax region with forceps. Termites were then placed into a PDA slant and the slant was incubated for up to 6 days at ambient temperature (28–32 °C). PDA slants were chosen to encourage fungal rather than bacterial growth. Very little bacterial growth was observed, and none that originated from termites. Slants were examined daily to determine the presence, location and size of fungal colonies with respect to the termite. To avoid including contaminants in analyses, fungal colonies were only counted if original growth clearly started forming on slants from the termite rather than at another location; only 2 cultures were excluded for this reason. The area of each fungal colony was estimated daily.

Four parameters were analyzed: 1) Day the fungus first appeared: the time (in days) until the first appearance of fungi on cultured worker(s) and soldier(s) of a colony; 2) Proportion with fungus: the proportion of workers and soldiers from each colony that had fungus growing on them on the day of first appearance; 3) Day all showed fungus: the time (in days) when all workers and all soldiers of a colony had fungal growth and 4) Rate of fungal growth: for each termite, the area of fungal coverage was measured on the first and second day after fungus was visible and change in area between these days was calculated. Growth was not measured later because fungus had reached the sides of the agar slants, making measurement unreliable. All parameters were compared using paired tests (pairing workers and soldiers from a colony) on proportions (parameter 2) or within-colony averages (parameters 1, 3 and 4). A paired t-test was used if data were normally distributed and a Wilcoxon signed ranks test if they were not.

### Comparison of fungal growth in the presence of combinations of workers and soldiers

Termites were collected from trails of 17 colonies as above and left with their trail material in clean plastic cups for 4–10 hrs before use. Termites were sorted and surface sterilized as above. They were placed into sterile 1.7 ml microfuge tubes in one of the following 8 combinations: 1W, 5W, 1W1S, 5W1S, 5W5S, 1W5S, 5S, 1S. Each colony was used once for each combination. Fifty µl of sterile 0.1% Tween 80 was added to the tubes. Termites were mashed using sterile microfuge pestles, the tubes were allowed to stand for 10–15 minutes for a pellet to form. After pellet formation, a sterile, 1 µl inoculating loop was used to transfer a total of 5 µl of the supernatant (approx. 10% of the total supernatant) to PDA slants. This protocol was chosen to minimize dilution of terpenoids placed onto slants as much as possible. Slants were checked every 24 hrs for fungal growth.

Each combination of workers/soldiers differed in total mass but the total liquid in tubes was held constant. Thus, the supernatant in the different combinations contained different concentration of worker/soldier derived nutrients, conidia and soldier secretions. Hypothesis 1 predicts that fungal growth increases with increasing termite biomass (i.e., increasing concentration of termite derived nutrients and conidia). Thus, based on a 3:1 mass ratio (workers to soldiers), termite combinations were expected to have the following *relative* rates of first appearance from slowest to fastest: 1S (1 mass unit), 1W (3), 1W1S (4), 5S (5), 1W5S (8), 5w (15), 5W1S (16), 5W5S (20). If hypothesis 2 is supported (i.e., soldier secretions inhibit fungal growth), two factors should affect fungal growth: first, growth should be inhibited by the presence of soldiers. This inhibition could be all-or-none or could increase with increasing soldier (antifungal) concentration. Second, growth should increase with an increase in worker number in the absence of soldiers because workers do not produce terpenoids. Thus, workers serve as a control to determine growth in the absence of soldier secretions. If hypothesis 2 is supported, the relative rate of appearance of fungi from slowest to fastest is expected to be: 5S, 1S, 1W5S, 1W1S, 5W5S, 5W1S, 1W, 5W (assuming that antifungal activity increases with concentration). The hypotheses were tested visually by graphing the mean first appearance of fungal growth for each of the 8 combinations according to the expected growth rate. The hypotheses were tested statistically by conducting pairwise comparisons using paired t-tests between specific combinations for which predictions of the two hypotheses were explicit. Pairwise combinations tested were 1W vs. 5W (both hypotheses predict faster growth in 5W), 1S vs. 5S, 1W vs. 1W1S, 1W vs. 1W5S, 5W vs. 5W1S and 5W vs. 5W5S. For the latter 5 comparisons, Hypothesis 1 predicts faster growth in the second combination of each pair; Hypothesis 2 predicts the opposite, in the case of 1S vs. 5S, no difference is predicted. These pairwise comparisons were also used to determine whether there is a threshold effect for fungistatic activity. The Bonferroni method was used to correct for the number of comparisons made for each combination (Zar 1984).

**Table 1.**
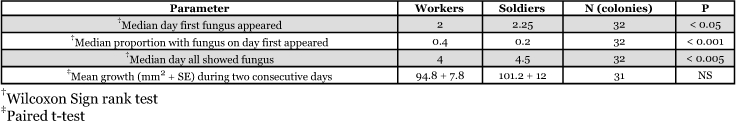
Fungal appearance and growth rate among workers and soldiers of *Nasutitermes*

**Table 2.**
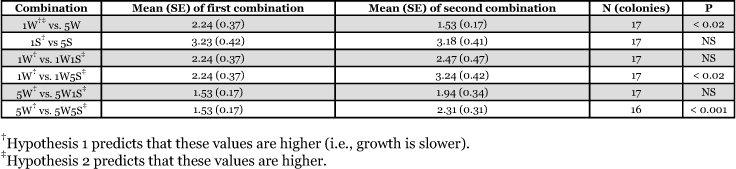
Mean (SE) days until appearance of fungal growth for pairwise comparisons for various soldier-worker combinations.

## Results

### Experiment 1: Comparison of fungal growth in the presence of 1 soldier *vs*. 1 worker

Fungus appeared significantly earlier on workers than on soldiers (Wilcoxon Sign Rank Test, Z = 2.26, P < 0.05; Table 1) and a greater proportion of workers showed fungal growth on the first day fungus appeared (Z = 4.25, P < 0.001; Table 1). Fungus grew from all except 5 workers and 4 soldiers within 6 days. However, excluding those 9 termites, it took significantly longer for fungal growth to appear on soldiers than on workers (Z = 3.12, P < 0.005; Table 1). Once fungus began to grow, there was no difference in the rate of growth (Paired t = 0.45, P > 0.5; Table 1).

### Experiment 2: Comparison of fungal growth in the presence of combinations of workers and soldiers

**Figure 1.**
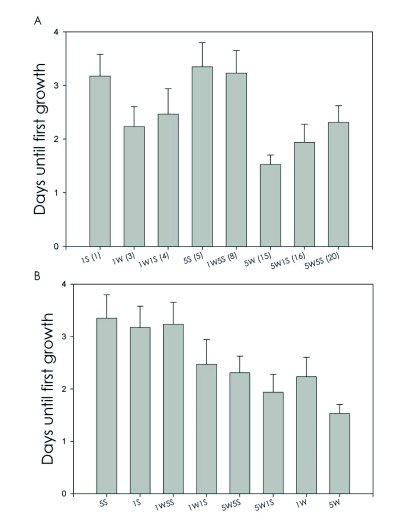
Relative growth rates of cultures based on predictions from hypotheses 1 and 2. Days until first growth of fungi in cultures containing a mixture of workers and soldiers. (A) Tests of the hypothesis that growth appears earlier in animals with greater biomass and/or exposure (hypothesis 1): growth should appear earlier as the X axis “increases” if Hypothesis 1 is supported. (B) Tests of the hypothesis that growth appears earlier in animals that lack frontal glands (hypothesis 2): growth should appear earlier as the X axis “increases” if Hypothesis 2 is supported.

Figure 1a shows that there is no obvious relationship between biomass/exposure and appearance of fungal growth, therefore hypothesis 1 (fungal growth increases with termite biomass) was not supported. However, Figure 1b shows an apparent negative relationship between the presence of soldiers and appearance of fungal growth, supporting hypothesis 2 (soldiers inhibit fungal growth).

All pairwise comparisons were in the predicted direction that supports hypothesis 2 (Table 2). Three of these comparisons were statistically significant: 1W vs 5W (P < 0.02), 1W vs 1W5S (P = 0.02), and 5W vs 5W5S (P < 0.001). Adding one soldier to either 1W or 5W did not significantly inhibit fungal growth, but adding 5S to either 1W or 5W did (Table 2), suggesting a threshold level between 1 and 5 soldiers for inhibition to occur.

## Discussion

In this study, soldier clearly inhibited growth of naturally-occurring fungi. On average, fungal colonies were first detected in cultures with soldiers a day later than in cultures with workers only. Although the specific components that inhibit growth were not tested, it seems likely that inhibition is caused by frontal gland secretions. Moore (1964, 1968) examined the chemical composition of the frontal gland secretions of six *Nasutitermes* species. The secretions consisted primarily of α-pinene, β-pinene and limonene. Rosengaus et al. 2000 found that both α-pinene and limonene caused inhibition of conidia germination of an entomopathogenic fungus (*M. anisopliea*), but α-pinene was most effective. In the current study, once growth appeared, there was no difference in growth rate of fungi between workers and soldiers. This may be due to the volatility of pinene and limonene (Moore 1964). If spores are inhibited from germination rather than actually killed, inhibition may disappear once the terpenoids evaporate.

Because entire termites were used in this study, it is possible that factors other than terpenoids were partly responsible for antifungal activity. For example, leaf-cutter ants culture bacteria that suppress growth of a pathogenic fungus that attacks their fungal gardens (Currie et al. 1999). However, suppression of fungal growth by bacteria seems likely to be of minor importance in the current study. First, leaf-cutter ants have visible patches of cultured bacteria on their cuticles (Currie et al. 1999); no such bacterial cultures have been observed on nasute termites. Second, almost all microbial growth observed in this study was fungal (R. Humber, USDA ARSEF, personal communication, 2004 samples). Finally, there was no direct evidence such as inhibition zones or overgrowth by bacteria that bacteria suppressed fungi. It is also possible that soldier secretions other than frontal gland terpenoids suppressed fungal growth. However, only components of frontal glands are known to have antifungal activity (Rosengaus et al. 2000). In addition, other functions of the other major glands are well documented: food processing (mandibular gland) and trail-laying (sternal gland) (Noirot 1969a, Noirot 1995).

Evidence was found of a threshold number of soldiers needed for inhibition in experiment 2. Although fungal growth was inhibited by fluids from a single soldier in the absence of workers, growth was not inhibited if worker fluids were combined with those of a single soldier. However, inhibition did occur in the presence of workers if their fluids were combined with those of 5 soldiers. Only 10% (5 µl) of the termite fluids were transferred to PDA medium. Thus, the equivalent of a single termite soldier may be enough to inhibit some fungal growth. It is unclear how these threshold levels relate to those found by Rosengaus et al. (2000). The authors used relative proportions of α-pinene and limonene consistent with those found in frontal glands; however, conidia were exposed to a much higher terpenoid amount in total than would be found in the frontal gland of a group of soldiers. Even so, the lowest concentrations used by Rosengaus et al. (2000) did not significantly inhibit germination, whereas one soldier alone, in the absence of a worker did affect growth in the present study. This suggests that frontal gland secretions may have greater biological activity than their chemical components alone or that *N. acajutlae* has highly potent frontal gland secretions.

It was also found that soldiers inhibited growth of a wide variety of fungi. These have been identified as belonging to the genera *Aspergillus, Cladosporium, Geotrichum, Paecilomyces, Penicillium*, and, *tentatively, Botryotrichum* and *Pyrenochaeta* (R. Humber, pers. comm.). Of these, *Aspergillus* and *Paecilomyces* may be entomophagous; however, most of the identified fungi are not pathogenic. Further testing is required to determine their exact relationship with *N. acajutlae*. However, it is interesting to ask why soldiers inhibit a wide variety of fungi (this work) but workers do not accrue net survival benefits in the presence of entomopathogenic fungi (Rosengaus et al. 2000). It may be that the primary benefit of fungistatic activity is suppression of competitive fungal growth at food sources rather than inhibition of pathogens in the nest. Relationships between termites and saprophytic fungi may take several forms. Termites in the Termopsidae and Rhinotermitidae prefer wood that has been “conditioned” by a variety of saprophytic fungi and can be attracted to such wood (Batra and Batra 1979; Noirot and Noirot-Timothee 1969). However, other termites are repelled by saprophytic fungi and some of these fungi are toxic to them (Batra and Batra 1979; Becker and Lenz 1976; Noirot and Noirot-Timothee 1969; Sands 1969). Perhaps *Nasutitermes* soldiers suppress fungal growth to avoid such toxins. Moreover, some xylophagous termites, including some nasutes, prefer intact dead wood, that hasn't been conditioned by fungi (Becker and Lenz 1976). For these termites, fungi may simply represent competitors for limited wood resources and secretions may serve to reduce competition.

The role of nasute soldiers is proving to be very wide-reaching. Their use of chemical defenses to combat small predators (e.g., ants) has long been known (Noirot 1969b). More recent evidence suggests that soldiers also deter larger predators (e.g. lizards) because they are distasteful (Fuller et al. 2003). Cephalic secretions may also inhibit pathogens (Rosengaus et al. 2000) and microbial competitors for wood. The genus *Nasutitermes* is the most specious of all termites (Kambhampati and Eggleton 2000). Perhaps the versatility of soldier defense compounds contributes to this success.
